# Avicenna and clinical experiences in Canon of Medicine

**DOI:** 10.25122/jml-2021-0246

**Published:** 2022-02

**Authors:** Farzaneh Ghaffari, Majid Taheri, Azam Meyari, Yasin Karimi, Mohsen Naseri

**Affiliations:** 1. School of Traditional Medicine, Shahid Beheshti University of Medical Sciences, Tehran, Iran; 2. Trauma and Injury Research Center, Iran University of Medical Sciences, Tehran, Iran; 3. Medical Ethics and Law Research Center, Shahid Beheshti University of Medical Sciences, Tehran, Iran; 4. Department of Persian Medicine, School of Medicine, Hamadan University of Medical Sciences, Hamadan, Iran; 5. Department of Persian Medicine, School of Medicine, Shahed University, Tehran, Iran; 6. Traditional Medicine Clinical Trial Research Center, Shahed University, Tehran, Iran

**Keywords:** Avicenna, clinical experiments, Canon of Medicine

## Abstract

Avicenna used his medical knowledge and experience of scientists from different nations to create a new style in medicine. For this reason, his textbook, Canon of Medicine, has been considered a medical reference in all universities worldwide for centuries. In this article, some valuable and interesting diagnostic and therapeutic clinical experiences mentioned in the Canon of Medicine are described in five sections. This research was conducted to review Avicenna’s specific clinical observations and interventions in PubMed, Google Scholar, and Scopus databases using the keywords “Avicenna” and “Canon of Medicine”. In this article, we presented several examples of diagnostic and therapeutic clinical experiences mentioned in the Canon of Medicine in 5 areas, including semiology, therapeutic strategy, urology, neurology, obstetrics, and gynecology. Canon of Medicine, as a complete medical series containing the medical experiences from different nations and Iranian medical scientists, has influenced the world’s medical knowledge for several centuries. Some of Avicenna’s clinical and experimental views can be useful from both a historical point of view and new research.

## Introduction

**Figure 1.**
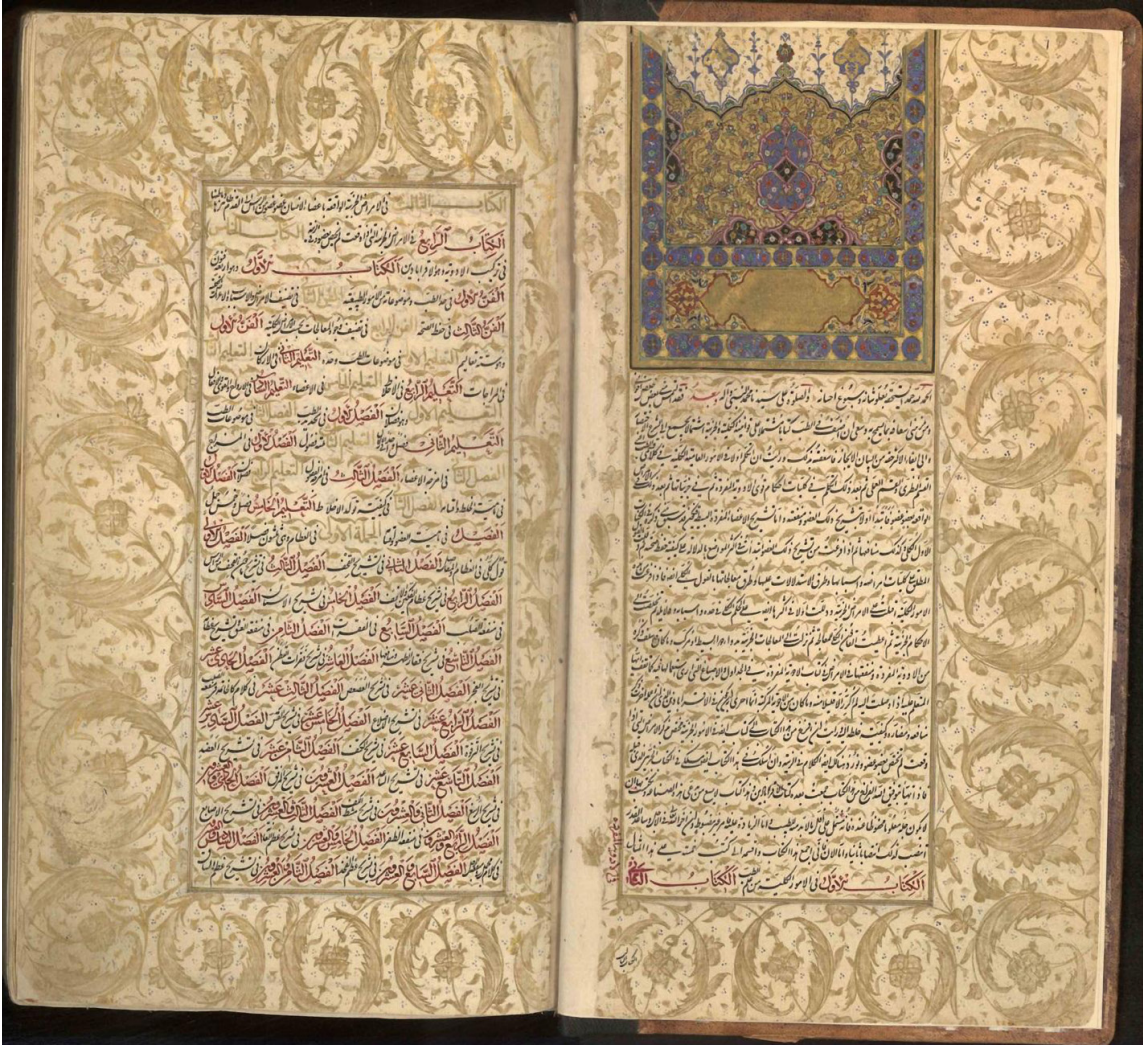
Cover page of The Canon of Medicine, Islamic Consultative Assembly Library, Tehran, Iran.

Avicenna (980–1037 AD) was born in a town near Bukhara called Afshaneh. He had considerable expertise in philosophy and logic in childhood and began to study medicine at the age of ten. His constant effort to learn from scholars of different nations and to think deeply about the basis of medical sciences and extensive clinical experiences and his unique memory and accuracy were some of the main reasons making him prominent among many other physicians [1]. His miraculous talents led him to become a famous physician at sixteen [2–4]. At the age of eighteen, he successfully treated the king of the Samanid dynasty in his time, Nuh ibn Mansour. This allowed him to access the royal library and the written medical science of all scholars and increase his knowledge in various fields [5, 6]. But his knowledge went beyond his time and geography. A few decades later, his most important medical book, the Canon of Medicine (Figure 1), was translated into Latin by Gerard of Cremona, and his worldwide fame spread. The medical curriculum of the Christian universities, including those in the British Isles, was also based on Avicenna’s writings until the mid-17^th^ century [7, 8]. Canon of Medicine became so popular that it was translated into Latin dozens of times in the West [9]. The Canon of Medicine is considered the most inﬂuential work on the healing arts during history [8]. According to Sir William Osler, no book in the history of medicine had been able to retain its value for centuries despite medical advances [10].

Undoubtedly, empiricism has been an important factor in the fame and permanence of his book. Avicenna stated that he reached indescribable horizons of healing science at the age of 16 due to the multiplicity of treatments and clinical experiences [11]. In other words, despite his extraordinary intelligence and constant effort, he introduced numerous patient visits and clinical experiences as the main factors of his scientific progress in the field of medicine. So certainly, one of the reasons that no book has been able to replace the Canon of Medicine in the world’s universities for five centuries is the clinical experiences that this physician expressed in the field of diagnostic methods and therapeutic interventions in this book.

In this study, we aimed to address a small number of Avicenna’s clinical observations and experiences. Some of Avicenna’s clinical experiences are discussed in five areas of semiology, therapeutic strategy, urology, neurology, obstetrics and gynecology and oncology by reviewing “Canon of Medicine” and articles related to “Avicenna” in Pubmed, Scopus, and Google scholar databases.

### 1: Semiology

1.1: Percussion: Leopold Auenbrugge (1722–1809 AD) is known as the father of percussion [12] in clinical examination. However, in the Canon of Medicine, percussion is mentioned for differential diagnosis of intestinal obstruction and Ascites. Avicenna stated in intestinal obstruction, a tympanum sound is heard when you hit the abdomen by finger, while in Ascites, whenever we hit the patient’s abdomen, we do not hear any sound, and succussion splash is positive [13].

1.2: Pregnancy diagnosis through pulse examination: Pulsology, from Avicenna’s point of view, is an important and helpful diagnostic method in specific diseases or physiological conditions. Therefore, he addressed this issue in a separate book. The multiplicity of clinical examinations in the absence of para clinical tools led Avicenna to understand the rules of pulsation in pregnancy diagnosis [14]. In the following centuries, advances in diagnostic tools led scientists to discover that pregnant women had different pulses [15]. Changes in pulse wave velocity have proven to be reliable in predicting pregnancy-induced hypertension [16].

### 2: Therapeutic strategy, especially nutrition therapy

2.1: Lifestyle medicine: In modern medicine, however, scientists were forced to reintroduce lifestyle medicine into medical literature about a millennium after Avicenna in 1989 due to the growing prevalence of chronic diseases [17], the vacancy of this therapeutic attitude is still quite noticeable in the curriculum of medical students [18, 19].

From Avicenna’s point of view, in the Canon of Medicine, a treatment has 3 basic steps. The first, most important, and least complicated step is lifestyle modification, especially nutrition. In addition to nutrition, management of physical activity and resting, evacuation and retention, sleep and wakefulness, psychological and mental control, air, water, and environmental factors are the other lifestyle modification issues. The second and third steps are drug therapy, manual therapy, and physiotherapy such as cupping and leech therapy, respectively [13].

Food therapy and nutrition have a long history in ancient Iran. Persian physician, Melanpus (400 BC), described food fortification by adding iron to the soldiers’ food [20]. Avicenna believed that nutrition had a critical role in human health and disease treatment. Therefore, he considered nutrition therapy the most important treatment method, including fasting, food restriction, and food modification.

2.2: Calories restriction: Today, the effect of calorie restriction has been approved by the medical community in managing various diseases such as diabetes mellitus, cardiovascular disease, malignancies etc [21]. Avicenna used the calorie restriction method to treat some patients [22]. Based on this theory, a study evaluated the effect of a short-term low-calorie diet on chronic sciatica, and the results showed that this intervention was significantly effective [23].

### 3: Urology:

3.1: Bladder function: The bladder function has two separate stages called the storage and voiding phases. These stages were explained by Yoshimora and Chancellor in recent decades. Avicenna, however, mentioned these two phases in bladder physiology a long time ago without modern facilities and equipment. In addition, his remarks on the histology, anatomy, and physiology of the urinary system, such as the anti-vesicoureteral reflux mechanisms in the bladder, show the depth of his experimental studies and those of physicians before him [24].

3.2: Urinary stone disease: According to modern epidemiological studies, Avicenna reported bladder stones mostly at an early age [25]. He also described the inversion therapy method and Credé maneuver to treat retention caused by bladder stones and also described the bladder surgery methods used for large bladder stones. Details of urinary stones surgery, such as the size of the incision and different approaches for different people, are given in the Canon of Medicine [24].

3.3: Catheterization and chemical lithotripsy of bladder stone: Avicenna has mentioned the correct position and lubricant material for catheterization for different purposes. According to Avicenna, one of the special and effective treatments for bladder stones is to use a catheter called “Zaragha” to deliver the crushing compounds directly to the bladder [13, 24].

### 4: Neurology

4.1: Facial palsy: Facial palsy is divided into central and peripheral categories. Clinical examination is very important in differentiating between central and peripheral types [26]. According to historical documents, Avicenna was the first physician to distinguish between central and peripheral facial palsy in his scientific works, indicating his expertise in clinical examination [27].

4.2: Therapeutic effect of electric waves: Considering the advancement of science, the effect of electric waves has been studied in various forms to treat neurological diseases these days. The effectiveness of this treatment has been proven in many diseases [28, 29]. Interestingly, Avicenna also used this method to treat some diseases such as epilepsy, despite all the scientific limitations [7, 30].

4.3: Spinal trauma: In the Canon of Medicine, the anatomy of the cervical, thoracic, lumbar, sacral, and coccygeal vertebrae is described according to what is classified in modern anatomy. In the chapter addressing spinal trauma, Avicenna described the possible side effects of injury to any part of the spine and various methods for treatment, including systemic and topical medications, calorie restriction, cupping, and even surgery. He also used spine traction in various ways to treat neurological complications of a displaced vertebra [31]. Today, the positive effect of this method was shown in some studies in the treatment of spinal problems, such as improvement of symptoms in herniation disc and cardiogenic dizziness [32, 33].

### 5: Obstetrics and Gynecology

5.1: Cervical lesions diagnosis: He used the speculum to study cervical diseases. According to historical evidence, he was apparently the first physician to use a mirror to reflect light into the area for a closer examination by the speculum [13, 34].

5.2: Cervical and vaginal mass surgery: Avicenna considered surgery the only treatment for infertility due to obstruction of the birth canal following enlargement of the mass in this area. The details and techniques of this type of surgery were described in his book [13].

5.3: Delivering hydrocephalic fetuses: This Iranian physician stated that if the delivery process was disrupted due to fluid accumulation in the fetus’ head (hydrocephalus) and an urgent action was needed, the head fluid should be drained using a special knife protected by fingers [13, 35].

## Discussion

In this article, some examples of clinical experiences in five fields of medicine and the position of empiricism from Avicenna’s point of view are discussed (Table 1). Utilization of lifestyle in prevention and treatment, differential diagnosis of central and peripheral facial palsy, medical and surgical treatment of spinal trauma, chemical lithotripsy and performing difficult surgeries to remove bladder stones considering the complex anatomy of the area, and cervical examination using speculum and the use of mirrors to enhance the view of the region, all indicate that Avicenna and Canon of medicine were many years ahead of their time.

**Table 1.** Some of Avicenna’s clinical experiments in the Canon of medicine.

**Medical branch**	**Topic**	**References**
**Semiology**	Percussion for differential diagnosis of intestinal obstruction and ascites	Avicenna [13]
	Pregnancy diagnosis through pulse examination	Avicenna [14]
**Therapeutic strategy**	Lifestyle medicine specially nutrition therapy	Avicenna [13]
	Calorie restriction	Nozad [22]; Safari [23]
**Urology**	Storage phase and voiding phase of bladder function	Madineh [24]
	Correct position and lubricant material for bladder catheterization	Madineh [24]
	More occurrence of bladder stone at early age	Changizi [25]
	Chemical lithotripsy of bladder stone	Avicenna [13]; Madineh [24]
**Neurology**	Differential diagnosis of central and peripheral facial palsy	Resende [27]
	Therapeutic effect of electric waves	Kelishadi [7]; Vakili [30]
	Medical and surgical treatment of spinal trauma	Ghaffari [31]
**Obstetrics and Gynecology**	Cervical lesions diagnosis	Avicenna [13]; Kadıoğlu [34]
	Cervical and vaginal mass surgery	Avicenna [13]
	Delivering hydrocephalic fetuses by surgery	Avicenna [13]; Khajavi Shojaei [35]

Empiricism has been so important that even medical education in Iran was based on this method. Haly Abbas (949–982 AD) [36], whose book Kamel al-Sinaa al Tibbiya was used for many years as a reference book in medical schools, believed that medical students should reside in hospitals and clinics in order to gain the necessary knowledge by visiting patients with their professors [37]. Also, the importance of clinical experience in the books of Rhazes (865–925 AD), as another prominent Iranian physician, is quite clear in presenting case reports and animal studies. He is even one of the pioneers in presenting clinical trial results with the control group in the history of medicine [38]. The high attention of trained physicians in clinical observations in this medical school is very important. For example, Abul Hasan Tabari (916–986 AD) was the first physician to discover the role of Sarcoptes scabiei in scabies. In his examinations, he concluded that very small worms are involved in the pathogenesis of this disease [39].

On the other hand, from Avicenna’s point of view, medical rationalism forms another important part of medicine. Avicenna believes that rational general rulers and the use of general rules for predicting minor cases can develop medical horizons. A second-century Roman physician, Galen also recommended empiricism and rationalism to medical students. He wrote a treatise titled “That the Physician must be a Philosopher” [40].

With the spread of “evidence-based medicine” from the 1990s, empiricism emerged as the mainstay of medical science, and the attention of rationalism was greatly diminished [41, 42]. For this reason, practical observations and experiences in medicine became more and more important [40]. As stated by various examples, empiricism through observations and clinical interventions has been an irreplaceable principle in education and treatment in Persian Medicine (PM). It seems that rationalism and medical theories of Iranian physicians have resulted from many clinical experiences in different historical periods, and the clinical richness of PM increased from generation to generation through an experimental-based educational method. It should be noted that today some researchers believe that giving importance to rationalism as a complementary method can further improve the performance of future physicians rather than relying solely on evidence from randomized controlled trials, meta-analyses etc [38, 39]. This idea is in line with the educational strategy in Iranian hospitals over the centuries. The strategy resulted in training prominent physicians such as Avicenna, Rhazes, Haly Abbas, and Abul Hasan Tabari.

## Conclusion

There is ample evidence in the Canon of Medicine that shows many of Avicenna’s achievements and leadership in medicine was because of innovative approaches in treatments, extensive clinical experience and efforts to find new physical examination methods to diagnose diseases. The diagnostic and therapeutic ideas expressed in this book appear to be based on clinical evidence that Avicenna himself or other Iranian physicians before him observed in their patients. Therefore, Persian medicine and evidence-based medicine can be linked by designing studies to test these experiences today.

## Acknowledgements

### Conflict of interest

The authors declare no conflict of interest.

### Authorship

FZ contributed to conceptualizing and editing the manuscript, MT contributed to editing the manuscript. AM contributed to writing the original draft. YK contributed to writing the original draft and data collection. MN contributed to data curation and methodology.
